# Clinical Approach to Evaluation of Underlying Cardiac Device Infection in Patients Hospitalized with Bacteremia

**DOI:** 10.14797/mdcvj.1271

**Published:** 2023-08-01

**Authors:** Dierdre B. Axell-House, Sarwat Khalil, M. Rizwan Sohail

**Affiliations:** 1 Division of Infectious Diseases, Houston Methodist Academic Institute, Houston Methodist Hospital, Houston, Texas, US; 2 Center for Infectious Diseases Research, Houston Methodist Research Institute, Houston, Texas, US; 3 Section of Infectious Diseases, Department of Medicine, Baylor College of Medicine, Houston, Texas, US

**Keywords:** cardiac implantable electronic devices, bacteremia, hematogenous seeding, infective endocarditis

## Abstract

More than 400,000 cardiac implantable electronic devices (CIEDs), including permanent pacemakers, implantable cardioverter-defibrillators, and cardiac resynchronization therapy devices, are implanted every year in the United States (US). Infection is a serious complication of CIED therapy and is associated with high morbidity and mortality. While CIED pocket infection can be diagnosed based on clinical exam findings, positive blood culture may be the only manifestation of CIED lead infection. Thus, management of bacteremia in patients living with CIEDs requires special consideration. This review summarizes contemporary data in the context of the recently updated 2023 Duke-International Society for Cardiovascular Infectious Diseases Criteria for Infective Endocarditis. We have synthesized these data into an algorithmic approach to streamline the diagnostic evaluation of CIED infection in patients presenting with bacteremia.

## Introduction

The term cardiac implantable electronic devices (CIEDs) includes permanent pacemakers, implantable cardioverter-defibrillators, and cardiac resynchronization therapy devices. Every year, more than 400,000 pacemakers and implantable cardioverter-defibrillators are implanted in the US.^1^ Infection is a serious complication after CIED implantation, affecting from 5% to 20% of patients.^2,3^ The incidence of CIED infections is increasing far beyond the increase in CIED prevalence over the past few decades.^2,4,5^ Infection is associated with high morbidity, and mortality in 10% to 30% of patients, more frequently in patients with systemic infections.^6,7,8^ The substantial cost of care for a patient with CIED infection is upwards of $100,000 to $300,000 per patient for bloodstream infections requiring complete CIED system explant as part of management.^9,10^

There are two distinct mechanisms of CIED infection: device or pocket contamination at the time of implantation or subsequent manipulation (direct inoculation),^11^ and hematogenous seeding of the CIED leads from remote sources of bacteremia. Device contamination is associated with early-onset (within weeks to months post-implantation) and often presents clinically as a generator pocket infection that may also be complicated by a bloodstream infection. Hematogenous seeding is associated with late-onset (typically greater than 6 months post-implantation) bloodstream infection. In this scenario, patients may have an initial episode of bacteremia unrelated to the CIED and later have relapsing bacteremia with evidence of CIED lead infection. The source of bacteremia occurring in patients with CIEDs may be CIED pocket infection, CIED-related infective endocarditis (CIED-IE), a non-CIED source, or unknown/cryptic. While evidence is clear for the management of patients with established diagnoses of CIED pocket infections or CIED-IE, the management of patients with CIEDs with bacteremia from cryptic or non-CIED sources is less established. Understanding the risk of hematogenous seeding of CIEDs during bacteremia is crucial to optimal diagnostic evaluation and management of CIED infections.

## Device Seeding Risk Varies by Pathogen

Once a blood culture drawn from a patient with a CIED shows bacterial growth, the patient’s morbidity and mortality are impacted by (A) whether infection of the CIED is the source of bacteremia, and (B) if the CIED is not the source of the bacteremia, whether the circulating bacteria will adhere to device leads and increase the risk of future CIED infection. The source of infection impacts management in terms of antibiotic choices and treatment duration and, more importantly, whether the patient will need to undergo an invasive procedure for device removal.

### Bacteremia due to Gram-Positive Organisms

The risk of hematogenous device seeding depends on the identity of the infecting pathogen. The majority (70% to 90%) of CIED infections are caused by gram-positive bacteria (GPB), with *Staphylococcus aureus* and coagulase-negative staphylococci being the most common pathogens.^12^ *S. aureus* is the most common cause of bacteremia in patients with CIEDs,^13^ with an estimated 30% to 50% of *S. aureus* bacteremia (SAB) associated with device infection.^14,15,16^

Sohail et al. generated a model to predict CIED infection in patients presenting with SAB but without signs of pocket infection. They found that the independent risk factors for CIED infection were having a pacemaker device, having undergone > 1 device-related procedure, and a duration of SAB ≥ 4 days.^17^ The study indicates that patients without these high-risk features can be managed with antibiotics and close monitoring without the need for immediate device extraction.

The risk of hematogenous seeding CIEDs after SAB can be further evaluated in patients who do not undergo CIED removal who then have relapsing SAB. In a longitudinal study of SAB in patients with CIEDs, 158 patients with CIEDs had SAB without evidence of CIED infection. Of these patients, 125 patients did not have CIED removal and survived the initial SAB episode, with 25 patients (20%) experiencing SAB relapse within 6 months. Twelve (48%) of the relapsed patients had evidence of CIED infection at re-presentation.^18^ Increasing duration of bacteremia was associated with an increasing risk of relapse.^18^ Another smaller study found a relapse rate of SAB due to new CIED infection was 36.7% in 30 patients who did not have any evidence of CIED infection during their initial SAB episode and did not have a CIED explant.^19^

Retrospective analysis of patients with CIED infection is also useful for examining seeding risk. In a study of patients with CIED infections, 21% of patients with a new diagnosis of CIED infection due to *S. aureus* had an episode of SAB in the preceding 12 months, whereas only 4% of non-*S. aureus* CIED infections had a preceding bacteremia.^20^ *S. aureus* is a significant cause of morbidity and mortality in patients with CIEDs, and mounting evidence indicates that CIEDs should be removed in the setting of SAB even if the source is clinically ambiguous. Indeed, in a study of SAB in patients without clear CIED pocket infection or CIED-IE, SAB was associated with increased hazard of death at 1 year if CIED extraction was not performed.^18^

Coagulase-negative staphylococci (CoNS) are the second most common cause of bacteremia in patients with CIEDs and are the most common cause of CIED-related infection overall.^12^ Determining which patients presenting with CoNS bacteremia have underlying CIEDs infection requires judicious appraisal as patients with CIEDs have increased risk of lead infection from CoNS due to biofilm formation. However, positive blood cultures with CoNS may represent contamination of blood cultures during collection since they are part of skin flora. With consideration for the patient’s history of illness and physical exam, a single positive blood culture for CoNS out of two or more sets may constitute a contaminant,^21^ but two separate positive blood cultures would constitute a major Duke-International Society for Cardiovascular Infectious Diseases (ISCVID) Criteria for infective endocarditis in the setting of CIED.^22^ Madhavan et al. conducted a study of non-*S. aureus* gram-positive cocci bloodstream infections occurring in patients with CIEDs: Of 44 patients with CoNS infections, 16 (36%) had CIED infections. Nineteen patients without evidence of CIED infection at index admission did not have empiric CIED explant and survived the initial infection, although five (26%) developed relapsing CoNS bacteremia within 12 weeks. No patient had evidence of CIED infection at the time of relapse; one patient’s relapse was attributed to a central venous catheter, two to a left ventricular assist device (LVAD), and two patients had unidentified sources.^23^ A more recent study examined 64 CIED patients with CoNS bacteremia, 41 (64.1%) of whom had definite or possible CIED infection. Of the 23 patients without initial CIED infection, one patient had a relapse of CoNS bacteremia in which the initial infection was due to central venous catheter, and the relapse was found to be CIED infection.^24^

Enterococci, particularly *Enterococcus faecalis* (Efs), remain among the top pathogens causing infective endocarditis^22^ and also cause CIED-related infection. Enterococci caused an estimated 4% to 5% of bacteremia attributed to CIED infection in a recent CIED registry study, with 14% of patients having CIED-related infection and 86% of patients having infective endocarditis.^25^ In a study of all Efs bacteremia occurring in patients with CIEDs in Sweden, 72 patients were identified and 5 were diagnosed with infective endocarditis. Of the 68 patients without evidence of CIED infection at the time of Efs bacteremia, 67 patients did not have CIED removal, and 7 (10%) of these patients had recurrence of Efs bacteremia. The source of Efs in the recurrent infections was unknown in five patients, urinary tract infection in one patient, and infective endocarditis in the setting of an aortic graft infection in one patient.^26^ Other less frequent causes of GPB in patients with CIED include streptococci, *Corynebacteria spp*., and *Cutibacterium acnes*.^27^

### Bacteremia due to Gram-Negative Organisms

The clinical importance and management of gram-negative bacteremia (GNB) in patients with CIEDs is gaining interest. GNB account for 6% to 10% of all bacteremia in patients with CIEDs.^13,27,28^ When evaluating the risk of CIED seeding during GNB and relapse, one early study showed that out of 52 patients with CIED and GNB, 3 were diagnosed with central venous catheter infections with no concomitant evidence of CIED infection but later developed recurrent bacteremia with evidence of CIED infection (2 *Pseudomonas aeruginosa* and 1 *Acinetobacter baumannii*).^27^

In another study of 49 patients with CIEDs and GNB, one patient had an initial infection that was a possible non-pocket CIED infection with *Serratia marcescens*, and 37 patients had no evidence of CIED infection, survived, and did not undergo device extraction. Two of the 37 patients later developed recurrent bacteremia (with *Klebsiella pneumoniae*), which in both cases was not attributable to CIED infection.^29^ In another study analyzing cardiac devices overall (including patients with CIEDs, LVADs, prosthetic valves, and multiple devices), the pathogens with the highest rates of definite device infection out of all episodes of GNB were *Pseudomonas aeruginosa* (53.8%) and *Serratia marcescans* (46.7%). This demonstrates an increased propensity of *Pseudomonas* and *Serratia* amongst the gram-negative pathogens to seed and cause infections of CIEDs and cardiac devices.^30^

A recent assessment by Chesdachai and colleagues of all GNB in patients with CIEDs (excluding patients with LVADs) evaluated 126 patients; of these, 4 had definite CIED infection and 10 had possible CIED infection. A frequency of 45.5% of *Serratia marcescens* bloodstream infections were attributed to CIED infections as the source, compared to 15% of *Pseudomonas aeruginosa* bloodstream infections and just 6.3% of all other GNB. None of the patients with definite CIED infection were able to have the device removed due to poor patient condition for surgical intervention. Among those four, two had *Pseudomonas aeruginosa* and one had *Serratia marcescens* infection; they died within 12 weeks of initial infection. The remaining patient had *Cardiobacterium hominis* and survived while maintained on chronic antibiotic suppression through 12-week follow-up. In the 10 patients with possible CIED infection, no one had CIED removal, one had *Pseudomonas aeruginosa* (died before 12-week follow-up), and four had *Serratia marcescens* (one died before 12-week follow-up). The other five patients with possible CIED infections with other GNB survived.^31^ Thus, it appears that the risk of CIED seeding with non-*Pseudomonas* non-*Serratia* GNB is quite low.

## Diagnostic Imaging for CIED Infection in the Setting of Bacteremia

Crucial to the management of bacteremia in CIED patients (beyond consideration of the pathogen) is determining whether there is active CIED infection or not. CIED infections can be divided into (1) CIED pocket infections, and (2) systemic infections typically manifesting as endocarditis with device lead and/or heart valve vegetations. CIED pocket infections are often easier to detect by signs of local inflammation at the generator pocket site and systemic symptoms are typically absent.^32^ Presence of systemic CIED infection/endocarditis has long been assessed by use of transthoracic and especially transesophageal echocardiogram. However, due to the limitations of echocardiograms in ascertaining a true lead infection, interest is growing in the use of labeled white blood cell scans and ^18^F-fluorodeoxyglucose positron emission tomography with computed tomography (PET-CT) to confirm or exclude CIED lead infection.

The diagnosis of endocarditis is critically important, as its management necessitates prolonged antibiotics, close follow-up of cardiologic parameters, and possible surgical intervention. Although echocardiography has been the mainstay of endocarditis diagnosis, transesophageal echocardiography (TEE) is superior to transthoracic echocardiography in the identification of pacemaker lead and other CIED-associated vegetations. The sensitivity of TEE for detecting vegetations is estimated at approximately 90% to 95%, while the sensitivity of the transthoracic echocardiogram is estimated at approximately 20% to 40%.^33,34,35^ However, TEEs are limited in their inability to detect the difference between an infected vegetation and a noninfected echodensity. Patel et al. conducted a study of patients with CIEDs who underwent TEE for noninfectious indications, and 33 patients (17.3%) were found to have lead-based echodensities. On follow-up (between 353 and 378 days), no patients developed signs of endocarditis or CIED infection.^36^ A more recent matched study compared 25 patients with CIED lead vegetations and infective endocarditis (IE) to 25 patients without infection who had incidental lead echodensities detected on TEE. Two blinded echocardiologists correctly diagnosed infection in 6 of 25 cases (24%), had low sensitivity for correct diagnosis (31-37%), and had low overall inter-reader agreement.^37^ No size or morphological characteristics were found to be discriminatory between infectious and noninfectious echodensities in either study.^36,37^ This highlights the difficulties in practical use of the long-standing most-accurate imaging technique for the diagnosis of IE and the need for alternative approaches.

The accuracy of TEE diagnosis of CIED-related infective endocarditis is dependent on clinical suspicion of infection and pre-test probability and is not intended to be used as a screening test. However, the possibility remains of identification of ambiguous echodensities in the setting of early, cryptic, or otherwise undetected endocarditis. Thus, there is interest in alternate imaging modalities, such as PET-CT, to diagnose CIED-related infective endocarditis. A meta-analysis in 2019 estimated sensitivity and specificity of PET-CT for CIED-related infective endocarditis to be 76% (95% CI, 65% to 85%) and 83% (95% CI, 72% to 90%) respectively. Estimated sensitivity and specificity were much higher at 96% (95% CI, 86% to 99%) and 97% (95% CI, 86% to 99%) respectively, for the diagnosis of CIED pocket infections.^38^

In terms of cardiac implants generally, PET-CT demonstrably improves sensitivity in detection of prosthetic valve endocarditis^39^ as well as in patients with other non-native cardiac material.^40^ Based on this evidence, PET-CT is now a major criterion as part of the new Duke-ISCVID criteria for infective endocarditis.^22^ Potential reasons for lower estimations of diagnostic accuracy for CIED-IE are primarily based on the small size of leads and vegetations, low spatial resolution of PET-CT imaging, motion artifact, and high metabolic activity (thus labeled fluorodeoxyglucose uptake) of the myocardium. Benefits of PET-CT over echocardiography and other imaging modalities include the ability to detect pulmonary septic emboli and other potential disseminated infections, such as osteomyelitis.^41^

Tagged white blood cell scans use either ^111^Indium-oxine (^111^In-oxine) or ^99m^Technetium-hexamethylpropyleneamine oxime (^99m^Tc HMPAO) with single positron emission computed tomography/computed tomography. The newer ^99m^Tc-HMPAO radiolabel has replaced ^111^In-oxine mainly due to lower radiation requirement, lower cost, and faster time to imaging.^42^ The only study of tagged white blood cell scans in CIED infections, performed by Erba et al., found that ^99m^Tc-HMPAO had an estimated 94% sensitivity and 100% specificity for CIED-associated infections (mixed pocket and lead infections) in a cohort of 63 patients.^43^ Lastly, cardiac CT is less sensitive for vegetations in infective endocarditis than TEE.^44,45^ However, it has a higher sensitivity for the identification of complications of infective endocarditis, such as perivalvular abscess, pseudoaneurysm, and valvular anatomical abnormalities.^45,46^ Thus, cardiac CT detection of IE or IE paravalvular complications is now a major criterion to the new Duke-ISCVID criteria for IE.^22^

## Management of Bloodstream Infections in Patients with CIEDs

Ultimately the importance of determining active CIED infection and stratifying the risk of hematogenous seeding of CIED is based on the need for complete CIED system removal to cure device infection. Cardiac device removal is critical to the successful cure of both pocket infections and CIED-infective endocarditis. One study of 416 patients demonstrated that prolonged antibiotics without device removal in patients with CIED infections was associated with a 7-fold increase in 30-day mortality after multivariable analysis.^47^ When presented with a new-onset bloodstream infection in a patient with CIED, subsequent management is dependent on the pathogen and the identification of active infection by imaging.

*S. aureus* is the most frequent cause of bacteremia in patients with CIEDs, and SAB is associated with high morbidity and mortality in these patients. As with any other pathogen, any clinical presentation consistent with CIED pocket infection should result in CIED removal. In the presence of SAB, patients with CIEDs should undergo TEE to evaluate for infective endocarditis of the leads or valves. Since TEE is not 100% sensitive, in the setting of significant clinical concern for endocarditis, PET-CT should be performed if available. A 2022 study demonstrated that when using expanded criteria for the diagnosis of CIED infection, a much higher percentage of patients were diagnosed with definite CIED infection with *S. aureus*, and device removal in this group was associated with the best mortality benefit compared to possible and rejected CIED infection diagnoses.^48^ The expanded CIED infection definition in the study permitted the use of PET-CT imaging data as a diagnostic criterion, which is now included as a major criterion in the Duke-ISCVID criteria for IE.^22^

**Figure 1:**
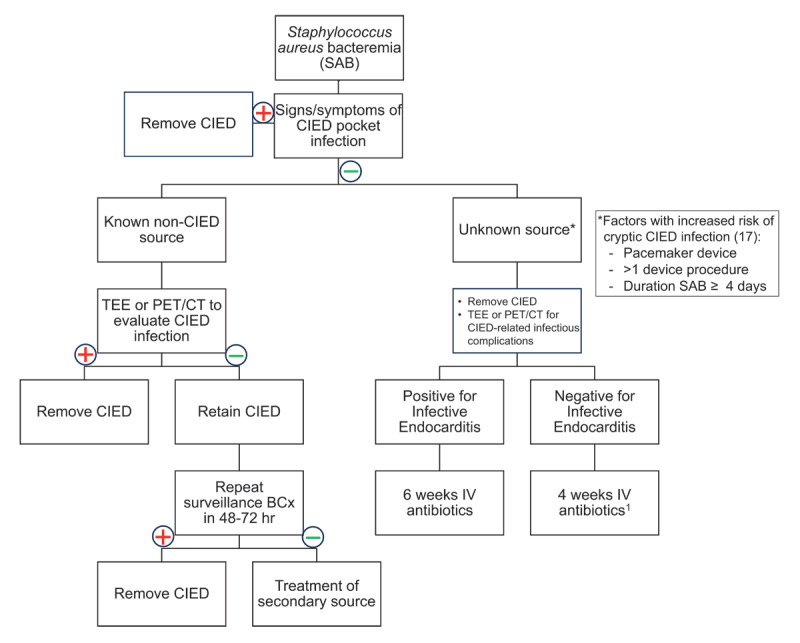
Management of *Staphylococcus aureus* bacteremia (SAB) in patients with cardiac implantable electronic devices (CIEDs). BCx: blood cultures; EOT: end of therapy; hr: hour; IV: intravenous; PET-CT: positron emission tomography-computed tomography; TEE: transesophageal echocardiography ^1^Duration may be extended to 6 weeks in presence of infectious complications requiring prolonged antibiotics such as osteomyelitis and endovascular infection (ie, septic venous thrombosis).

We present an algorithm for management of SAB in patients with CIEDs in Figure 1. Diagnosis with CIED-IE due to *S. aureus* is an indication for prompt CIED removal, as several studies describe a significant increase in mortality in this setting without CIED extraction or when delayed.^14,17,49^ Patients with IE in this setting also should undergo 6 weeks of targeted intravenous (IV) antibiotics, and subsequent follow-up imaging at the end of therapy. Given the potential for false negative imaging and the high morbidity and mortality, patients with SAB with unknown source and negative TEE and/or PET-CT should still have CIED removal and undergo 4 weeks of targeted IV antibiotics. For patients with a known non-CIED source of SAB with a negative TEE and PET-CT, treatment of the alternative source can commence, the CIED can be retained, and surveillance blood cultures repeated 48 to 72 hours after the prior blood cultures. If repeat blood cultures are positive, the patient is deemed to have persistent bacteremia and CIED removal should be pursued.

**Figure 2:**
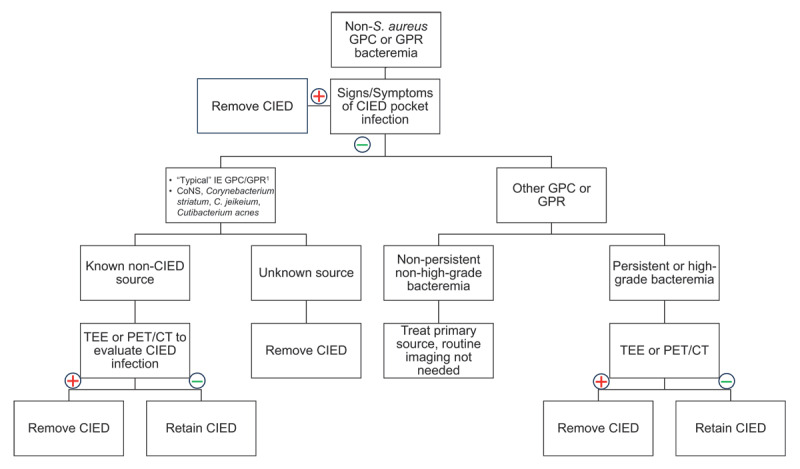
Management of non-*S. aureus* gram-positive cocci and gram-positive rod bacteremia in patients with cardiac implantable electronic devices. CoNS: coagulase-negative staphylococci; PET-CT: positron emission tomography-computed tomography; TEE: transesophageal echocardiography ^1^*Staphylococcus lugdunensis, Enterococcus faecalis*, streptococci except *S. pneumoniae* and *S. pyogenes, Granulicatella spp*., *Abiotrophia spp*., *Gemella spp*.

Besides *S. aureus*, the GPB coagulase-negative staphylococci and enterococci cause significant rates of bacteremia in patients with CIEDs. Although the frequency of other GPB is lower, several GPB have been added to the new Duke-ISCVID criteria as typical IE pathogens in patients with cardiac-related prosthetic material. These pathogens include coagulase-negative staphylococci, *Corynebacterium striatum, C. jeikeium*, and *Cutibacterium acnes*.^22^ Thus, two separate blood cultures positive for these organisms in patients with CIEDs, or other typical organisms that commonly cause IE (including *E. faecalis*), should be considered an indication to proceed with imaging to diagnose IE, such as TEE and potentially PET-CT (Figure 2). Other GPB causing brief bacteremia or with a known alternative source do not require TEE or PET-CT imaging. If a GPB that uncommonly results in IE causes a particularly high grade (from three or more blood culture sets) or persistent bacteremia, imaging of the CIED should be pursued at that time.

**Figure 3:**
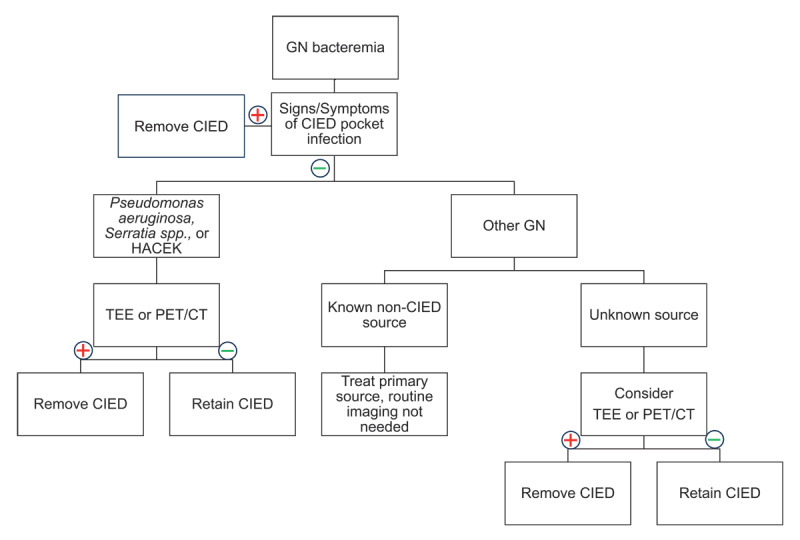
Management of gram-negative bacteremia in patients with cardiac implantable electronic devices. HACEK: *Hemophilus spp., Aggregatibacter spp., Cardiobacterium spp., Eikenella spp., Kingella spp*.; PET-CT: positron emission tomography-computed tomography; TEE: transesophageal echocardiography

GNB cause bacteremia in patients with CIEDs less frequently. However, the existing data indicates that bacteremia with either *Pseudomonas aeruginosa* or *Serratia marcescens* is an increased risk for CIED seeding and infection. Another consideration is the HACEK group organisms (*Haemophilus* species, *Aggregatibacter* species, *Cardiobacterium hominis, Eikenella corrodens*, and *Kingella* species), which are GNB at increased risk of causing IE generally although with low frequency overall. Detection of bacteremia with these organisms in patients with CIEDs should prompt imaging with TEE or PET-CT, particularly in the absence of a known secondary source (Figure 3). Separately, other GNB so infrequently seed CIEDs and cause CIED infections that if a non-CIED source is known, then no routine imaging of the CIED is necessary. If there is an unexplained persistent or high-grade GNB, then CIED imaging should be considered.

## Conclusion

The likelihood of a patient presenting with bacteremia resulting in seeding of their CIED is dependent on the causative pathogen. While outward signs and symptoms may be sufficient for diagnosing CIED pocket infections as the source of a bacteremia episode, cardiac imaging is necessary in cases of systemic presentations to evaluate for underlying CIED-IE for some organisms. The 2023 Duke-ISCVID criteria for IE recently had several updates in major and minor imaging criteria, allowing for greater sensitivity for diagnosis of IE overall, including in patients with CIEDs. Imaging should be pursued for all patients with SAB with CIED, and CIED removal should be pursued or strongly considered in nearly all cases. The decision to remove CIED in other cases of GPB or GNB is dependent on bacterial identity and the likely source of infection. An algorithmic approach to diagnostic evaluation of bacteremia in patients with CIEDs may streamline management decisions and improve overall patient outcomes.

## Key Points

- The optimal approach to management of bacteremia in patients with cardiac implantable devices (CIEDs) is dependent on bacterial identity, with different considerations for *Staphylococcus aureus*, non-*S. aureus* gram-positive bacteria, and gram-negative bacteria.
- When imaging is indicated to identify potential CIED infection as the source of bacteremia, positron emission tomography with computed tomography should be considered in addition to transesophageal echocardiography.
- The 2023 Duke-International Society for Cardiovascular Infectious Diseases Criteria for Infective Endocarditis include new data that will improve the diagnostic accuracy for CIED-related infective endocarditis.
